# Nickel quercetinase, a “promiscuous” metalloenzyme: metal incorporation and metal ligand substitution studies

**DOI:** 10.1186/s12858-015-0039-4

**Published:** 2015-04-23

**Authors:** Dimitrios Nianios, Sven Thierbach, Lenz Steimer, Pavel Lulchev, Dagmar Klostermeier, Susanne Fetzner

**Affiliations:** Institute of Molecular Microbiology and Biotechnology, University of Muenster, Corrensstrasse 3, Muenster, D-48149 Germany; Institute of Physical Chemistry, University of Muenster, Corrensstrasse 30, Muenster, D-48149 Germany

**Keywords:** Dioxygenase, Flavonol, Metalloprotein, Nickel, Coordination geometry, Cell-free protein synthesis

## Abstract

**Background:**

Quercetinases are metal-dependent dioxygenases of the cupin superfamily. While fungal quercetinases are copper proteins, recombinant *Streptomyces* quercetinase (QueD) was previously described to be capable of incorporating Ni^2+^ and some other divalent metal ions. This raises the questions of which factors determine metal selection, and which metal ion is physiologically relevant.

**Results:**

Metal occupancies of heterologously produced QueD proteins followed the order Ni > Co > Fe > Mn. Iron, in contrast to the other metals, does not support catalytic activity. QueD isolated from the wild-type *Streptomyces* sp. strain FLA contained mainly nickel and zinc. *In vitro* synthesis of QueD in a cell-free transcription-translation system yielded catalytically active protein when Ni^2+^ was present, and comparison of the circular dichroism spectra of *in vitro* produced proteins suggested that Ni^2+^ ions support correct folding. Replacement of individual amino acids of the 3His/1Glu metal binding motif by alanine drastically reduced or abolished quercetinase activity and affected its structural integrity. Only substitution of the glutamate ligand (E76) by histidine resulted in Ni- and Co-QueD variants that retained the native fold and showed residual catalytic activity.

**Conclusions:**

Heterologous formation of catalytically active, native QueD holoenzyme requires Ni^2+^, Co^2+^ or Mn^2+^, i.e., metal ions that prefer an octahedral coordination geometry, and an intact 3His/1Glu motif or a 4His environment of the metal. The observed metal occupancies suggest that metal incorporation into QueD is governed by the relative stability of the resulting metal complexes, rather than by metal abundance. Ni^2+^ most likely is the physiologically relevant cofactor of QueD of *Streptomyces* sp. FLA.

**Electronic supplementary material:**

The online version of this article (doi:10.1186/s12858-015-0039-4) contains supplementary material, which is available to authorized users.

## Background

**Scheme 1:**
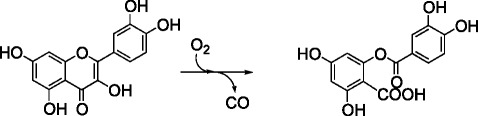
Conversion of quercetin to 2-protocatechuoylphloroglucinol carboxylic acid, catalyzed by quercetinase (flavonol 2,4-dioxygenase).

**Figure 1:**
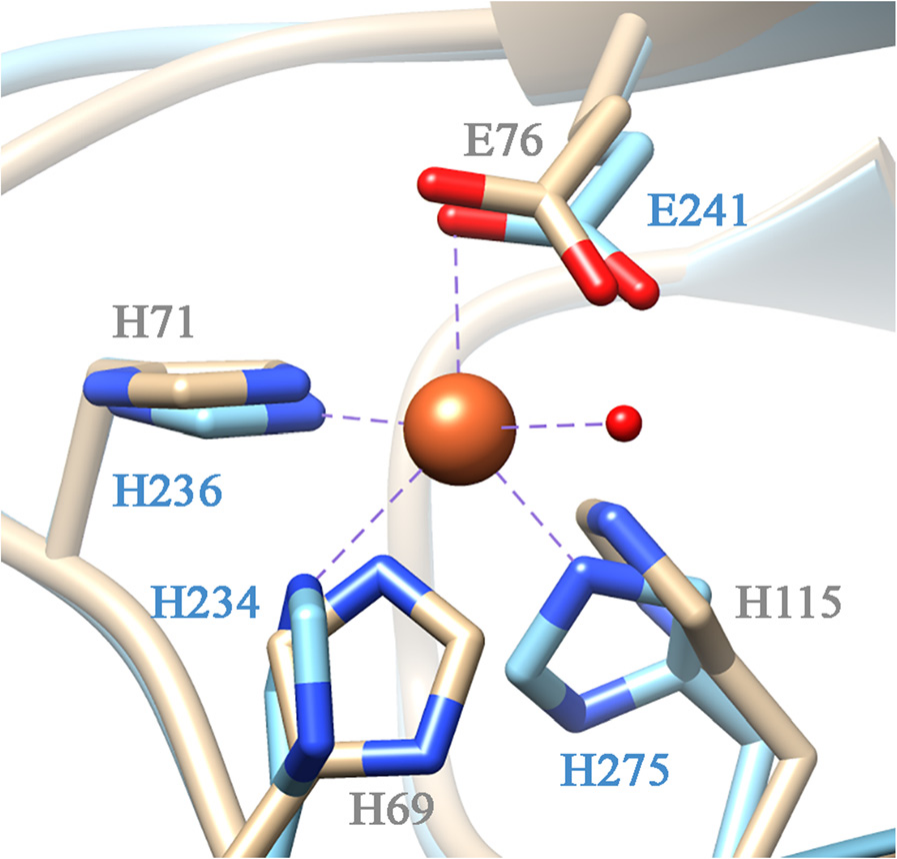
Model of the QueD metal center. The iron-containing *Bacillus subtilis* quercetinase [2] (PDB code 1y3t (chain B); residues indicated in blue) served as template for homology modelling of *Streptomyces* QueD (gray).

Quercetinase (flavonol dioxygenase, EC 1.13.11.24) catalyzes the 2,4-dioxygenolytic cleavage of quercetin, a flavonol that is produced by numerous plants, to form carbon monoxide and the depside (diphenolic ester) 2-protocatechuoylphoroglucinol carboxylic acid (Scheme 1). The bacterial and fungal quercetinases that have been characterized to date all belong to the cupin superfamily [1-4]. The cupin domain is characterized by a β-barrel fold which comprises two conserved motifs with the consensus sequences G(X)_5_HXH(X)_3,4_E(X)_6_G and G(X)_5_PXG(X)_2_H(X)_3_N. The conserved glutamate and the three histidine residues provide the ligands for a divalent metal ion in the active center of the enzyme [5] (Figure 1). Among the fungal quercetinases, the enzyme of *Aspergillus japonicus* has been characterized most intensively [1,6-9]. It is an extracellular glycoprotein with a bicupin scaffold, i.e., each subunit consists of two cupin domains. Two examples of bacterial quercetinases have been reported; both are cytoplasmic proteins. The enzyme of *Bacillus subtilis* consists of bicupin subunits that form a homodimer [2], whereas the enzyme of *Streptomyces* sp. FLA, which shows 35.9% sequence identity with the C-terminal cupin domain of *Bacillus* quercetinase, is a dimer of monocupin subunits [3,10].

The majority of dioxygenases require a metal cofactor for catalysis, which is most often a nonheme iron [11]. Interestingly, all the extracellular fungal quercetinases isolated so far rely on a mononuclear copper center for activity [1,4,12-14], whereas the cytoplasmic quercetinases of *B. subtilis* and *Streptomyces* sp. FLA appear to be promiscuous enzymes capable of using different metal ions for catalysis. *Bacillus* quercetinase has originally been purified from a recombinant *E. coli* host grown in LB (Lysogeny Broth) as an iron protein [2,15,16]. Reconstitution experiments suggested that Co, Cu, and Mn also support catalysis [2], and characterization of the kinetic parameters of the Mn- and Co-forms revealed that the catalytic efficiency was highest for the Mn-enzyme [17]. In contrast to *Bacillus* QueD, *Streptomyces* QueD, purified from recombinant *E. coli* cells grown with various metal ions, was found to be most active with Ni^2+^, which is highly unusual for oxygenases [3]. However, in this as well as in other studies on bacterial quercetinases, the proteins were heterologously produced in *E. coli*. As pointed out in a recent review, when using a high-expression heterologous system for the synthesis of Ni-QueD, “the relevance to metal speciation of protein in the native host remains unclear” [18].

*Streptomyces* sp. strain FLA grows very poorly on quercetin as sole carbon source [10]. Transcription of the *queD* gene of strain FLA is induced by quercetin, however, the regulatory mechanism remained elusive. An effect of Ni^2+^ on *queD* expression was not observed [19]. Because quercetin shows antibacterial activity, acting on multiple cellular targets [20-23], it is conceivable that a major physiological role of quercetinase is to detoxify the flavonol. The quercetin O-methylation, hydroxylation, or glycosylation reactions mediated by various *Streptomyces* spp. [24,25] may serve the same function.

In this study, we prepared different metal forms of *Streptomyces* QueD by *in vivo* and *in vitro* approaches, to identify the physiologically relevant metal cofactor(s), and to find out which factors determine metal selection. Replacement of individual residues of the 3His/1Glu motif gave insight into the significance of the amino acid ligands for metal occupancy, protein folding, and function.

## Results

### Metal selectivity and catalytic activity of recombinant QueD

**Figure 2:**
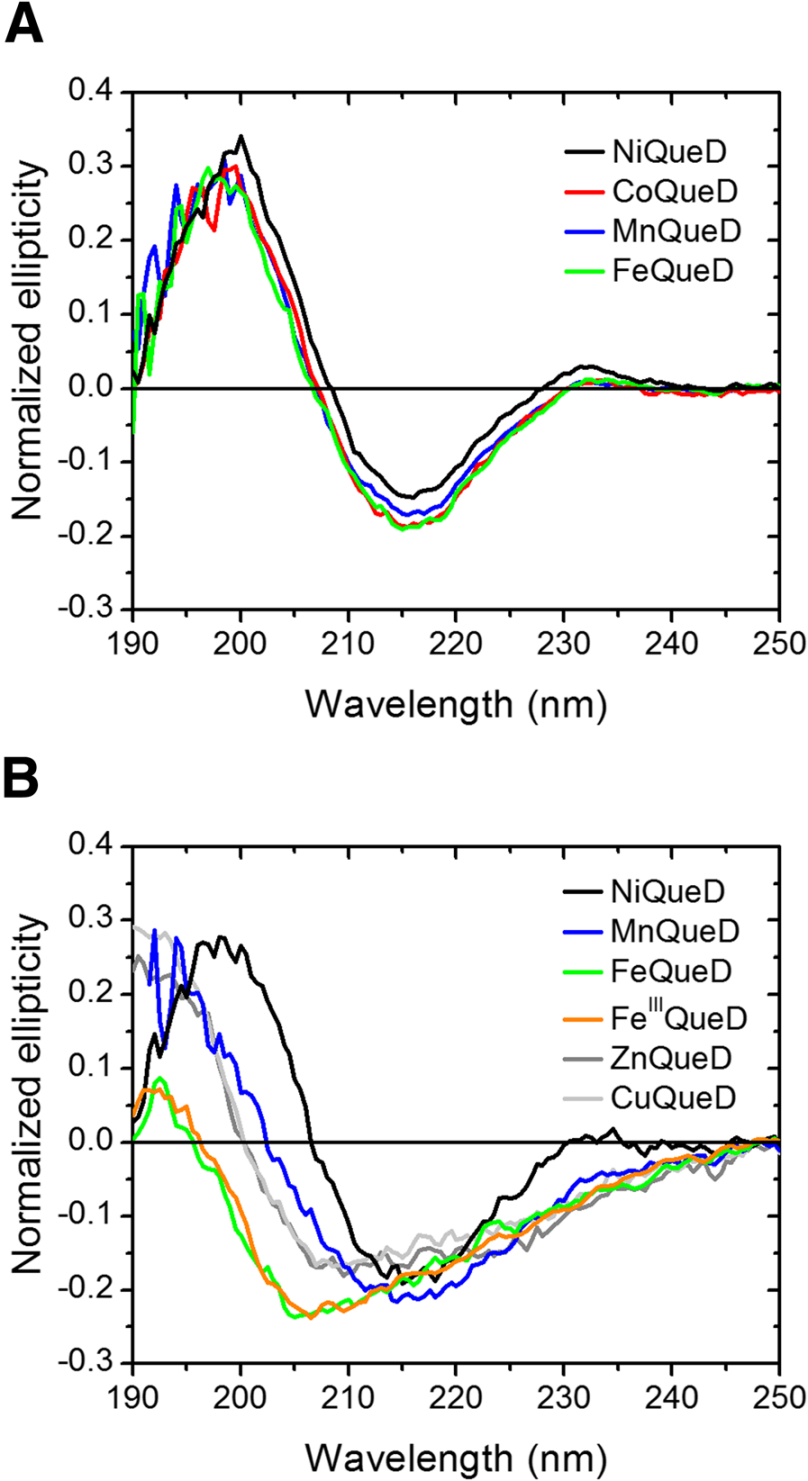
Far-UV CD spectra of QueD proteins produced *in vivo* and *in vitro.* **(A)**, Proteins purified from recombinant *E. coli* cells, and **(B)**, proteins synthesized *in vitro* by cell-free protein synthesis in presence of 1 mM Ni^2+^, Mn^2+^, or iron salts, or 0.2 mM Cu^2+^ or Zn^2+^. Protein samples in 10 mM potassium phosphate buffer (pH 8.0) were adjusted to ~10 μM (based on measuring absorbance at 280 nm). Spectra were recorded at 25°C in a 1 mm path length cell; bandwidth: 0.1 nm **(A)** or 0.5 nm **(B)**.

To enable a systematic study of the metal selectivity of QueD, as well as of the effects of different transition metal ions on quercetinase activity, we produced the enzyme recombinantly in *E. coli* which was grown in media supplemented with an excess of the metal ion of interest. Preparations of C-terminally Strep-tagged QueD, purified to electrophoretic homogeneity from cells grown in Ni^2+^-supplemented medium (Additional file 1: Figure S1), generally contained about 0.6 − 0.8 equivalents of nickel per protein monomer. Cobalt contents of QueD isolated from cells grown in CoCl_2_-supplemented medium varied significantly between the batches (0.5 to >1 equivalent per protein monomer). QueD protein produced by recombinant *E. coli* grown in Fe^2+^-supplemented medium showed varying iron contents in the range of 0.3 to 0.8 equivalents of iron per protein monomer. When QueD was purified from cells grown in medium supplemented with 30 μM MnCl_2,_ the proteins contained only between 0.2 to 0.5 equivalents of Mn and varying amounts of other metals, predominantly Ni, but also Fe and Zn. Increasing the concentration of MnCl_2_ in the cultivation medium to 1 mM did not increase the Mn occupancy of the recombinant protein. To assess the effect of the metal ion on the overall structure of the protein, the CD spectra of Ni-, Co-, Fe- and Mn-QueD produced *in vivo* were recorded. The spectra were very similar and characteristic of proteins that are predominantly composed of β-sheets (Figure 2A).

The catalytic activity of the recombinant proteins decreased in the order Ni-QueD > Co-QueD > Mn-QueD (Table 1). An estimation of the metal-related specific activities of QueD forms, i.e., the specific activities if QueD were fully occupied with the metal of interest (Additional file 2: Table S1), suggests that the iron form of QueD is not catalytically competent.

**Table 1:** **Metal contents and specific activities of selected batches of recombinant QueD proteins**

**Protein** ^**a**^	**Metal content (equivalents per protein monomer)**	**Specific activity (U mg** ^**−1**^ **)**
Ni-QueD	0.70 Ni, 0.20 Fe, 0.03 Co, 0.06 Zn, 0.01 Mn	137
Co-QueD	0.51 Co, 0.07 Ni, 0.05 Fe, 0.02 Zn, 0.01 Mn, 0.01 Cu	30
Mn-QueD	0.44 Mn, 0.12 Zn, 0.08 Fe, 0.05 Ni, 0.01 Co, 0.01 Cu	18
Fe-QueD	0.72 Fe, 0.05 Ni, 0.02 Zn, 0.02 Cu, 0.01 Mn	8

^a^The designations of the QueD metal forms refer to the metal of interest and do not imply exclusive occupancy of the protein with this metal.

Metal ions which contribute to ≥0.01 equivalents per protein monomer are listed.

Characterization of a Zn-form of QueD would be very interesting from a mechanistic point of view, because the Zn^2+^ ion is redox-inert in biological systems. However, attempts to produce Zn-QueD by the *in vivo* approach resulted in protein preparations that contained nickel rather than zinc (up to 0.67 equivalents of Ni and between 0.1 and 0.26 equivalents of Zn per protein monomer).

Since fungal quercetinases depend on a Cu^2+^ center, it also would be interesting to characterize a Cu-form of the bacterial QueD. However, in the reducing environment of the *E. coli* cytoplasm, copper ions are in the Cu^1+^ state, and attempts to produce Cu-QueD by the standard expression system were not successful. Fusing QueD to PelB- or DsbA-leader peptides in order to secrete the recombinant QueD to the *E. coli* periplasm resulted in formation of intracellular rather than periplasmic protein, precluding the production of Cu-QueD by periplasmic Cu^2+^ incorporation.

In summary, the order of metal ions according to occupancy of recombinant QueD is Ni ≈ Co > Fe > Mn, while in terms of activity we observe the ranking Ni > Co > Mn.

### QueD from the wild-type strain, *Streptomyces* sp. FLA

The bacterial quercetinases described to date were all produced heterologously in *E. coli*. They were observed to be promiscuous with respect to metal ion incorporation, thus their natural cofactor(s) incorporated by the original wild-type strain remained unassigned [2,3,10,15-17]. Using a four-step protocol, QueD from *Streptomyces* sp. FLA was purified to approximately 91% electrophoretic homogeneity, as deduced from densitometric analysis of the Coomassie-stained gel (Additional file 1: Figure S1). According to ICP-MS (inductively coupled plasma – mass spectrometry) analysis, the protein preparation contained 0.33, 0.28, 0.16 and 0.02 equivalents of Ni, Zn, Fe and Cu, respectively, per monomer. We cannot exclude the possibility that the purification protocol enriches distinct metal forms of QueD, however, QueD activity eluted as distinct peaks from the columns. Because heterologously produced QueD can incorporate cobalt or manganese besides nickel and iron (see above), it is remarkable that the content of these metals was below detection, especially as the mineral salts medium used for growth of *Streptomyces* contained CoCl_2_ (8.4 nM) and MnCl_2_ (1.5 nM) besides NiCl_2_ (0.8 nM). The relatively high contents of zinc in the protein preparation were surprising. The growth medium was supplemented with 3.5 nM ZnSO_4_, but as cells accumulate metal ions to a different extent, zinc may be concentrated by a high factor. The protein preparation, which contained minor amounts of other proteins, showed a specific activity of 49 U mg^−1^ (Additional file 3: Table S2). Considering that recombinant QueD with a nickel occupancy of 33% should have a specific activity of about 60 U mg^−1^ (*cf.* Additional file 2: Table S1), the catalytic activity of wild-type QueD is likely mediated by a Ni^2+^ center.

### Cell-free protein synthesis (CFPS) of QueD in the presence of metal ions

From the analysis of QueD proteins produced *in vivo*, it is difficult to assess to which extent metal ion availability and/or intrinsic preferences of the protein determine metal incorporation. To exclude effects caused by the limited *in vivo* availability of free metal ions, we aimed at producing QueD using *in vitro* approaches. However, *in vitro* reconstitution experiments, following protocols used for other cupin proteins [2,26], resulted in precipitation of QueD. Therefore, we tested an *E. coli* cell-free coupled transcription-translation system for production of different metal forms of the QueD protein. There are relatively few studies on cell-free synthesis of metalloproteins in the literature. For example, successful synthesis by *in vitro* transcription-translation systems was reported for manganese peroxidase (a heme protein) [27], a ferredoxin [28], [FeFe] hydrogenase [29], and Cu,Zn-superoxide dismutase [30]. The yields reported were in the range of about 30 − 50 μg ml^−1^ [27,29,30] or lower and seldom reached several 100 μg of protein from 1 ml reaction [28]. Often only part of the total protein was functional [27,29].

In order to identify suitable conditions for CFPS in the presence of metal ions, we first used a QueD-EGFP fusion protein as reporter, and determined the relative protein level by measuring EGFP fluorescence during CFPS. At concentrations below or equal to 1 mM of Ni^2+^, Fe^2+^, or Mn^2+^, the relative EGFP fluorescence intensities in the coupled transcription-translation reactions were similar to those observed in CFPS reactions performed in the absence of additional metal ions (Additional file 4: Figure S2 A − C, F), suggesting that these metals do not significantly affect translational efficiency in general. In case of CFPS reactions in the presence of Ni^2+^ or Mn^2+^, the quercetinase activity in the samples increased with increasing metal concentration in the reaction mixtures (except for the reaction that contained 3 mM Ni^2+^) (Additional file 4: Table S3). Unfortunately, zinc and copper ions had an inhibitory effect on the efficiency of QueD-EGFP synthesis (Additional file 4: Figure S2 D − E), as also reported in CFPS studies on other metalloproteins [30,31]). The absence of quercetinase activity in the Cu^2+^- and Zn^2+^-CFPS samples (Additional file 4: Table S3) suggested that Cu^2+^ and Zn^2+^ are either not incorporated into the QueD-EGFP fusion protein, or do not support catalysis.

To purify (Strep-tagged) QueD proteins synthesized by CFPS, reactions were performed on a 1 ml scale in the presence of Ni^2+^, Mn^2+^, or Fe^2+/3+^, or Zn^2+^ or Cu^2+^, and the proteins were prepared by Strep-Tactin affinity chromatography. The isolation of QueD proteins was verified in Western blots of denaturing polyacrylamide gels (Additional file 5: Figure S4). Far-UV CD spectra were recorded to find out whether the proteins synthesized *in vitro* have adopted the same overall fold as those produced *in vivo* (Figure 2). The spectrum of QueD produced in Ni^2+^-supplemented CFPS reactions retained the characteristics of that of the Ni-QueD formed *in vivo*, with a negative band at about 215 nm and a positive band at 198 nm, indicating that QueD in the presence of nickel ions is able to fold to its native state *in vitro*. The protein produced in the presence of 1 mM Mn^2+^ retained a high β-sheet content. In contrast, the CD spectra of the proteins obtained from CFPS in presence of Cu^2+^ (0.2 mM), Zn^2+^ (0.2 mM), Fe^2+^ or Fe^3+^ (1 mM) differ significantly from that of the nickel enzyme. Thus, the metal ions tested can be ordered as Ni > Mn > > Zn, Fe, Cu according to their effect on secondary structure of QueD. Altogether, the findings suggest that while the efficiency of CFPS is similar in the presence of Ni^2+^, Mn^2+^ or Fe^2+^, in the *in vitro* situation only nickel ions support QueD folding to its native state*.*

QueD purified from CFPS reactions supplemented with Ni^2+^ and Mn^2+^ showed quercetinase activities of 21.3 U mg^−1^ and 6.6 U mg^−1^, respectively. For QueD isolated from CFPS-reactions supplemented with Cu^2+^, Zn^2+^, Fe^2+^ or Fe^3+^, the activity was approximately 0.1 U mg^−1^. Control experiments without metal ion supplementation indicated that such residual activity cannot be attributed to the supplemented metals, however, traces of Mn^2+^ and/or Ni^2+^ ions present in the *E. coli* extracts may confer activity to a subpopulation of the *in vitro* produced proteins. In terms of catalytic activity of QueD metal forms, we therefore obtain the ranking Ni > Mn.

### Replacement of the 3His/1Glu ligand amino acids of Ni-QueD

**Figure 3:**
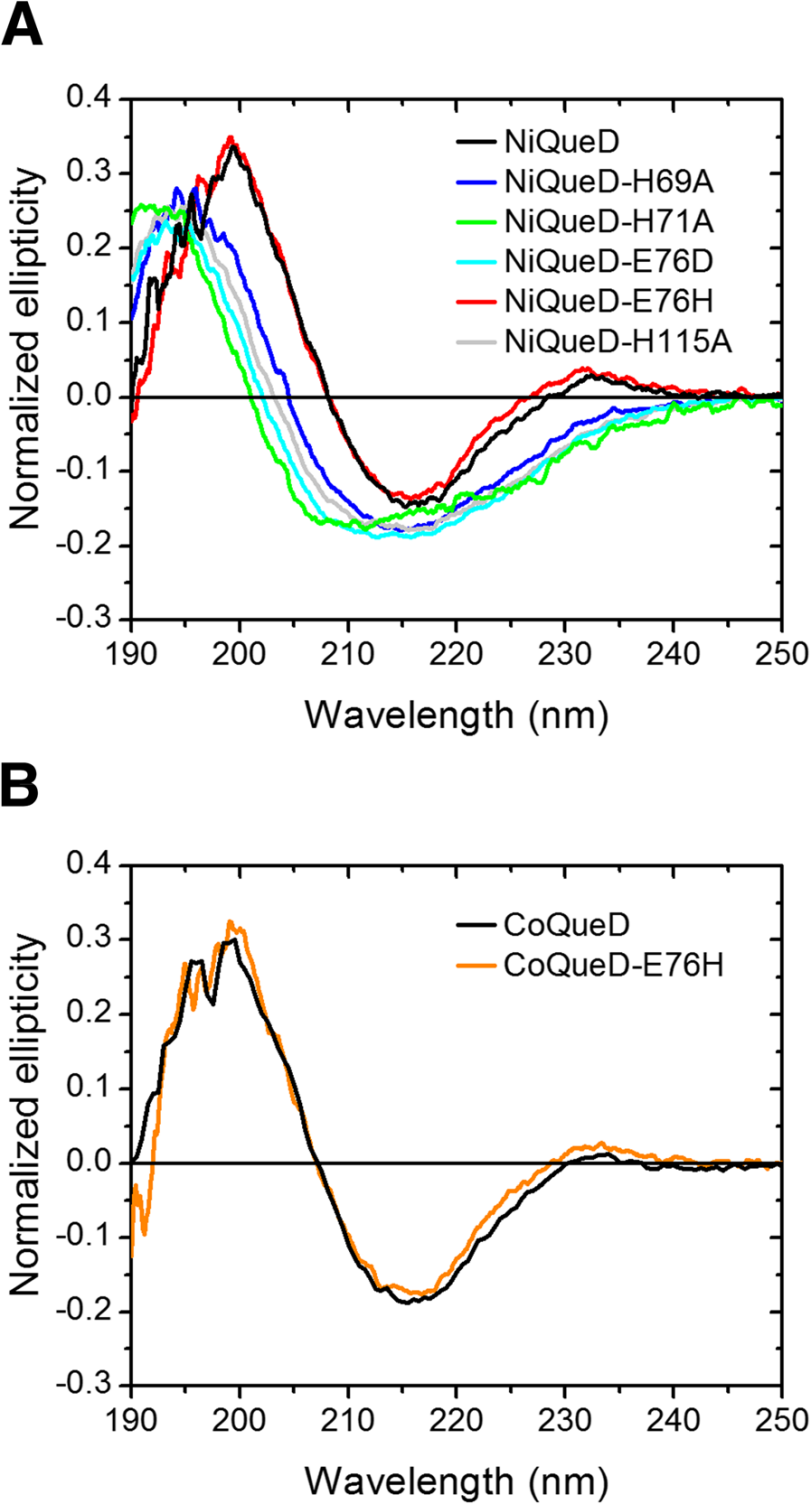
Far-UV CD spectra of QueD variants carrying amino acid replacements. **(A)**, Ni-QueD proteins with amino acid substitutions in the 3His/1Glu motif proposed to ligate the metal ion, and **(B)** Co-QueD with a substitution of the glutamate ligand by histidine. Protein samples in 10 mM potassium phosphate buffer (pH 8.0) were adjusted to ~10 μM (based on measuring absorbance at 280 nm). Spectra were recorded at 25°C in a 1 mm path length cell; bandwidth: 0.1 nm.

To analyze the role of the metal-ligating amino acids for the structural integrity and function of Ni-QueD, the individual residues of the 3His/1Glu motif (Figure 1) were replaced by site-directed mutagenesis, and the QueD proteins produced by the respective *E. coli* strain grown in Ni^2+^-supplemented medium were purified and characterized with respect to metal contents, catalytic activity, and ability to bind quercetin (Table 2). All variants, with the exception of the H69A substitution, showed a decrease in nickel contents compared to the wild-type protein, and a relative increase in the zinc contents pointing towards a somewhat relaxed metal specificity. The *K*_D_ values of the protein-quercetin complexes show that all protein variants retained a high affinity for the organic substrate. Replacement of the histidine residues at position 69 or 115 with alanine resulted in catalytically inactive protein variants, whereas the QueD-H71A and -E76D proteins retained marginal activity (Table 2). The CD spectra of the protein variants showed an increased intensity of the negative band (at approx. 215 nm) and a hypsochromic (blue) shift of the positive band (<200 nm) compared to the wild type protein (Figure 3A). Thus, the loss of activity could be due to perturbation of the secondary structure.

**Table 2:** **Metal contents and activities of QueD proteins carrying ligand replacements, and** ***K***_**D**_ **values of protein-quercetin complexes**

**Protein** ^**a**^	**Metal content (equivalents per protein monomer)**	**Spec. activity (U mg** ^**−1**^ **)**	***K***_**D**_ **(μM)**
Ni-QueD-H69A	0.88 Ni, 0.07 Fe	b.d.^b^	3.1 (0.9)
Ni-QueD-H115A	0.34 Ni, 0.29 Zn, 0.07 Fe, 0.02 Co, 0.01 Mn	b.d.	2.6 (0.6)
Ni-QueD-H71A	0.18 Ni, 0.16 Zn, 0.06 Fe	0.90 (0.04)	10.4 (2.9)
Ni-QueD-E76D	0.28 Ni, 0.24 Zn, 0.05 Fe	1.32 (0.01)	4.0 (1.0)
Ni-QueD-E76H	0.54 Ni, 0.03 Cu, 0.02 Fe, 0.01 Zn	3.44 (0.13)	1.3 (0.1)
Co-QueD-E76H	0.6 Co, 0.03 Cu, 0.02 Fe, 0.01 Zn, 0.02 Ni	0.19 (0.01)	n.d.^c^

^a^The designations of the QueD metal forms refer to the metal of interest and do not imply exclusive occupancy of the protein with this metal.

^b^b.d., below detection.

^c^n.d., not determined.

Metal ions which contribute to ≥0.01 equivalents per protein monomer are listed. For the specific activities and *K*_D_ values, the average of 3 experiments is given with the standard deviations in brackets. The *K*_D_ value of the complex of Ni-QueD with quercetin, determined for the preparation specified in Table 1, was 10.1 ± 2.9 μM.

Replacement of E76 by histidine resulted in a protein that showed about 2% of the activity of the wild-type protein and an about 8-fold decreased *K*_D_ value compared to that of the quercetin complex with Ni-QueD (Table 2). Interestingly, the CD spectrum of the Ni-QueD-E76H protein was nearly superimposable to that of Ni-QueD (Figure 3A), demonstrating that, in contrast to the other protein variants tested, the E76H variant retained the wild-type conformation. This was also observed for the cobalt form of this protein (Figure 3B). To verify that the mutant protein catalyzes the same reaction as wild-type QueD, the organic products of quercetin conversion by Co-QueD and Co-QueD-E76H were extracted and subjected to LC/MS analysis. The LC elution profiles were identical and both showed a main peak at a retention time of 18.2 min. ESI mass spectrometry of the corresponding peaks revealed ions at *m/z* 324.1 (100%) and 307.0 (22%) for [M + NH_4_]^+^ and [M + H]^+^, respectively. This corresponds to the previously observed mass for the molecular ion of the product of quercetin conversion by recombinant Ni- and Co-QueD and is consistent with formation of the depside 2-protocatechuoylphloroglucinol carboxylic acid (C_14_H_10_O_8_) [3]. The possibility that the same product could also result from a 2,3-dioxygenolytic side reaction to form an α-oxo acid, and subsequent hydrolysis to formate and the depside, was examined by determining the concentration of formate in enzyme assays. Previous analysis of the reaction of (His_6_-tagged) Ni- and Co-QueD had revealed an amount of formate corresponding to about 1.3% of the quercetin converted [3]. In the reaction of Strep-tagged Co-QueD, the formate detected corresponded to 2.3% (±0.2%) of substrate converted, whereas 3.0% (±0.8%) of side product was observed in the reaction of the Co-QueD-E76H protein. Thus, both the wild-type and the mutant Co protein mediate this side reaction to a similar extent.

The observation that Ni- and Co-QueD-E76H were the only proteins in the series of variants that retained the wild-type fold may suggest that the metal ion is coordinated by four amino acid ligands in these variants, i.e., in a 4His motif instead of the canonical 3His/1Glu motif. The HSAB concept predicts a higher stability of the Ni/His complex compared to a Ni/Glu complex, because the ligating nitrogen of His is softer than the ligating oxygen in Glu, leading to a larger stability of complexes with the soft Ni ion (or the Co ion). The E76H variant shows an increase in affinity for its electron-rich organic substrate, indicated by the low *K*_D_ value (Table 2). This effect might be due to the altered charge distribution around the metal, since a carboxylate ligand (as in E76) transfers more charge to the metal ion than an imidazole nitrogen. Alternatively, or additionally, subtle rearrangements of all the ligands around the metal may account for the decrease of the *K*_D_ value of Ni-QueD-E76H compared to that of the Ni-QueD-quercetin complex.

## Discussion

Bacterial quercetinases have been described to be able to use a defined set of different metal ions for catalysis, but the molecular basis of such metal selection is largely unclear. When using a recombinant high-expression system in *E. coli* for the production of *Streptomyces* QueD, the metal contents of the purified proteins suggested that QueD binds Mn^2+^, Fe^2+^, Co^2+^, and Ni^2+^ in the order Mn < Fe < Co ≈ Ni. This ranking is similar to the Irving-Williams series, which describes the stability of divalent transition-metal complexes as Mn^2+^ < Fe^2+^ < Co^2+^ < Ni^2+^ < Cu^2+^ > Zn^2+^ [32]. Interestingly, QueD purified from the wild-type *Streptomyces* sp. strain contained mainly nickel and zinc. However, quantitative metal analyses cannot distinguish between metal ions unspecifically adsorbed to the protein surface or those coordinated to the conserved metal-binding 3His/1Glu motif of the cupin protein.

Because the concentrations of “free” transition metal ions are thought to be in the 10^−7^ to 10^−15^ M range [33,34], metal incorporation into QueD in the *E. coli* expression system may be limited by metal ion availability. In contrast, the cell-free transcription-translation system allows for supplementation with defined metal ion concentrations. To outcompete divalent ions possibly brought in by the highly concentrated *E. coli* extracts used in the CFPS reactions, we added metal ions in excess, at concentrations which still supported protein synthesis (*cf.* Additional file 4). The CD spectra and activity data of the QueD forms produced *in vitro* indicated that intrinsic properties of the protein rather than metal ion availability govern the formation of native metalloenzyme, with Ni^2+^ as the strongly preferred ion.

The coordination geometry of the metal center should play a significant role in determining the preference of QueD for its active-site ion. Ni^2+^ centers with metal binding by a 3His/1Glu motif show a preference for octahedral geometry [35,36]. Co^2+^ and Mn^2+^ centers are usually also hexa-coordinated with an octahedral geometry [37]. Zn^2+^ typically has coordination numbers of four or five in active sites of enzymes, with a preference for tetrahedral geometry [37]. Cu^2+^ bound to proteins is typically coordinated by four ligands in a square planar geometry [37,38], or with an additional water ligand positioned to form a trigonal bipyramidal geometry [39]. Fe^2+^ complexes in proteins are either penta- or hexa-coordinated, with penta-coordination as most common [40]. Possibly, formation of native, catalytically competent (Ni-, Co-, Mn-) QueD correlates with the propensity of the metal ions to form an octahedral complex geometry, as dictated by the ligands that constitute the metal binding site of the protein. If so, the metal center of *Streptomyces* QueD would comprise two additional ligands (such as water molecules) besides the four amino acid ligands and thus would be different from that of copper-dependent *A. japonicus* quercetinase, which shows a penta-coordinated metal center with 3 His, 1 Glu and one water molecule [1]. Interestingly, an octahedral coordination sphere was also proposed for the catalytically highly active Mn-QueD of *B. subtilis* [17], whereas its less active Fe-form, which has been crystallized, is penta-coordinate with a square pyramidal or distorted trigonal bipyramidal geometry [2]. Thus, the geometry of the nickel center of *Streptomyces* QueD may be different from that shown in Figure 1 which is based on homology modelling using the crystal structure of quercetinase of *B. subtilis* as template.

The CD spectra of QueD proteins carrying replacements of individual residues of the 3His/1Glu motif suggested that all four residues are important for structural integrity of QueD, although Glu76 can be exchanged to histidine without affecting the secondary structure of the protein. The important structural role of both metal and ligands in QueD is in contrast to several other cupin proteins, which tolerate the substitution of individual metal-coordinating amino acid residues or which even retain their structure upon *in vitro* metal depletion [26,41,42].

## Conclusions

*Streptomyces* quercetinase (QueD), a metal dependent flavonol dioxygenase with a cupin fold, is rather promiscuous with respect to the incorporated metal ion. This study shows that QueD produced in a heterologous host requires Ni^2+^, Co^2+^ or Mn^2+^, i.e. metal ions that prefer an octahedral coordination geometry, for native secondary structure content and catalytic activity. Ni^2+^ ions support the formation of native QueD in a cell-free transcription/translation system. The *in vivo* and *in vitro* data suggest that metal ion specificity of QueD is determined by both chemical stability (the Irving-Williams series) and intrinsic geometric constraints dictated by the metalloprotein. Taking all criteria together, Ni appears to be the physiologically optimal ion for *Streptomyces* quercetinase. Crystal structures of QueD, as well as of QueD-E76H protein and enzyme-substrate complexes, would be most interesting to compare this bacterial quercetinase to the fungal copper quercetinase with atomic resolution.

## Methods

Research performed in this study does not involve human subjects, human material, or human data.

### Materials

Chemicals were purchased from Sigma-Aldrich, except for tRNAs from *E. coli* strain MRE 600 which were obtained from Roche Applied Science. Restriction enzymes, T4 DNA ligase, and other reagents for molecular biology were purchased from Thermo Scientific. DNA primers, listed in Table 3, were obtained from Microsynth AG (Balgach, Switzerland) or MWG Biotech AG (Ebersberg, Germany).

**Table 3:** **Oligonucleotides used for gene amplification and site-directed mutagenesis**

**Name**	**Nucleotide sequence (5′ → 3′)**	**Application**
quedfor	TGACCATCGAATACGCCACCCGTCACC	Amplification of *queD*
quedrev	GACGTCGTACTGCTCGGGCACGGTC	
strepfor	ATAAGAAT*GCGGCCGC*ATTAGAATGGAGCCACCCGCAGTTCGAAAAATGAGATCCGGCTGCTAACAAAGCCCGAAAGG	Exchange of His_6_-tag for StrepII-tag
streprev	ATAAGAAT*GCGGCCGC*AAGCTTCCTTCCCTCGATACTCCCGGTGTGC	
gfpfor	TAT*AAGCTT*GCCGGTGCGGAAAATC	Amplification of the *EGFP* gene
gfprev	TATA*GCGGCCGC*CTGGTACAGTTCATCCATG	
H69Afor	CCTCGCACGCGGACACCTACG	Exchange of His69 for Ala
H69Arev	CGGCGGGGATCACCTCGCCCTTG	
H71Afor	CGCCCACTCGGCCGCGGACACCTACGAGGTCTTC	Exchange of His71 for Ala
H71Arev	GGGATCACCTCGCCCTTGGGGCCCTCGCAG	
H115Afor	ATGGAACGCCACCACTCGCAGGTCG	Exchange of His115 for Ala
H115Arev	GCGGTAGGCGGCCACGCAGTTCTTCGGTACGAAGC	
E76Hfor/E76Dfor	CGTCTTCTACATCACCCAGGGC	Forward primer for the exchange of Glu76 for His or Asp
E76Hrev	TGGTAGGTGTCCGCGTGCGAGTGG	Reverse primer for the exchange of Glu76 for His
E76Drev	TCGTAGGTGTCCGCGTGCGAG	Reverse primer for the exchange of Glu76 for Asp

Recognition sites for restriction endonucleases are italicized.

### DNA techniques

Isolation of plasmid DNA from recombinant *E. coli* strains, extraction of DNA fragments from agarose gels, and purification of PCR products were carried out by the innuPREP kit series (Analytik Jena AG, Jena, Germany). Standard protocols were used for agarose gel electrophoresis, restriction digestion, and DNA ligation. Exchange of the His_6_-tag sequence in pET23a-*queD* [10] for a StrepII-tag sequence was performed by amplification of the plasmid with the primer pair strepfor/streprev (Table 3), restriction with DpnI and NotI, and circularisation with T4 DNA ligase. For fusion of enhanced green fluorescent protein to QueD, the *egfp* gene was amplified by PCR, using the primer pair gfpfor/gfprev (Table 3), and plasmid pET27b-*egfp* harboring a synthetic gene (GenScript) for EGFP (F64L/S65T/Q80R/K238Q) as template. The PCR product and pET23a-*queD*-*Strep* were restricted with *Hind*III and *Not*I, purified with the innuPREP Doublepure kit (Analytik Jena AG), and ligated, resulting in pET23a-*queD-egfp-Strep*. Transformation of *E. coli* strains DH5α and BL21 (DE3) [pLysS] with the constructed plasmid was performed as described by Hanahan [43]. Site-directed mutagenesis of the *queD* gene (GenBank accession number AM234612) was performed according to the protocol of the Phusion™ Site-Directed Mutagenesis Kit (Thermo Scientific) using pET23a-*queD-Strep* as template, Phusion™ High-Fidelity DNA polymerase for amplification, and the primers listed in Table 3. Confirmatory DNA sequencing of the insert and flanking regions of the plasmids was performed by GATC Biotech AG (Konstanz, Germany) or MWG Biotech AG.

### Cell-free protein synthesis (CFPS)

Cell-free protein synthesis was conducted at 30°C using the PANOx-SP system [44], except that instead of S30 cell extract, S12 extract from *E. coli* strain BL21 (DE3) was used, which was prepared as described by Kim *et al.* [45]. Crude extract of *E. coli* strain BL21 (DE3) containing T7 RNA polymerase was prepared separately as described in ref. [44]. In order to quantify the relative amounts of protein produced by CFPS, the plasmid pET23a-*queD-egfp-Strep* was used as template for *in vitro* transcription-translation reactions supplemented with metal salts (0.05 − 3 mM). The relative amount of EGFP present in the samples (as QueD-EGFP fusion) was determined by measuring the average fluorescence emission at 510 − 570 nm upon excitation at 490 nm. Cell-free synthesis of QueD-Strep, using pET23a-*queD*-*Strep* as template, was performed in the presence of 1 mM NiCl_2_, 1 mM MnCl_2_, 0.5 mM FeCl_2_ or 1 mM (NH_4_)_2_Fe(SO_4_)_2_ (optionally supplemented with 1 mM DTT), 1 mM FeCl_3_, 1 mM or 0.2 mM CuCl_2_, and 1 mM or 0.2 mM ZnCl_2_.

### Expression and purification of C-terminally Strep-tagged QueD proteins

*E. coli* BL21 (DE3) [pLysS, pET23a**-***queD-Strep*] as well as *E. coli* strains containing a mutant pET23a**-***queD-Strep* plasmid were grown at 37°C to an OD_600_ of 0.5 in M9 minimal medium [46] containing ampicillin (100 μg ml^−1^) and chloramphenicol (34 μg ml^−1^). Subsequently, metal salts (MnCl_2_, FeCl_2_, CoCl_2_, NiCl_2_, or ZnCl_2_) were added to the cultures to final concentrations of 10 μM (in case of Ni^2+^ and Co^2+^), 30 μM (for Mn^2+^, Fe^2+^, and Zn^2+^) or 1 mM (for Mn^2+^), and expression of *queD* was induced with 1 mM IPTG. The cultivation temperature was reduced to 25°C for 3 h and then shifted to 20°C for another 15 h. The cells were centrifuged (4°C, 10 min at 12,000 × *g*) and the cell pellets were stored at −80°C. For the preparation of cell extracts, cells were resuspended in 100 mM Tris/HCl buffer (pH 8) containing 300 mM NaCl and 1 mM MgCl_2,_ supplemented with 12.5 units of Benzonase (Novagen). After incubation on ice for 1 h, the suspension was sonicated and then centrifuged (4°C, 40 min at 38,360 × *g*). Recombinant QueD proteins were purified from cell extract supernatant by affinity chromatography on a 2 ml Strep-Tactin® column (IBA, Göttingen, Germany) using an FPLC system. QueD proteins synthesized by *in vitro* transcription/translation were isolated using a 0.5 ml Strep-Tactin® gravity column. After removing unbound proteins with 100 mM Tris/HCl buffer (pH 8.0) containing 300 mM NaCl, Strep-tagII fusion proteins were eluted with 2.5 mM desthiobiotin in the same buffer. Fractions containing the protein of interest were pooled, concentrated by ultrafiltration in a Vivaspin 20 concentrator (Sartorius Stedim Biotech), frozen in liquid nitrogen and stored at –80°C.

### Growth of *Streptomyces* sp. FLA and purification of QueD protein

Growth of *Streptomyces* sp. strain FLA in complex medium, followed by resuspension of the biomass in mineral salts medium supplemented with quercetin, was performed as described previously [10]. The growth medium contains ammonium ferric citrate (1 μM) and 100 μl/l of SL-6 trace element solution according to Pfennig [47], resulting in final concentrations of Cu^2+^, Ni^2+^, Mn^2+^, Zn^2+^ and Co^2+^ salts of 0.6 nM, 0.8 nM, 1.5 nM, 3.5 nM, and 8.4 nM, respectively. For the preparation of cell extracts, *Streptomyces* biomass was suspended in 50 mM Tris/HCl buffer (pH 7.0) and disrupted by sonication. After a centrifugation step (38,360 × *g*, 90 min) to remove cell debris, (NH_4_)_2_SO_4_ was gradually added to the cell extract supernatant to a final concentration of 0.4 M, and precipitated proteins were removed by centrifugation at 38,360 × *g* for 40 minutes at 4°C. The supernatant was applied to a 20 ml Phenyl-Sepharose CL-4B column (GE Healthcare) that was equilibrated in 50 mM Tris/HCl, pH 7.0, containing 0.4 M (NH_4_)_2_SO_4_. Quercetinase was eluted by stepwise reducing the concentration of (NH_4_)_2_SO_4_ (0.4 to 0 M) in the buffer. The protein fractions with quercetinase activity were pooled and dialyzed against 50 mM Tris/HCl buffer (pH 8.0). The protein pool was applied to a 6 ml Q Sepharose Fast Flow column (GE Healthcare) equilibrated in 50 mM Tris/HCl buffer (pH 8). Protein fractions with quercetinase activity were eluted with 0.3 M NaCl and dialyzed as described above. A second anion exchange step was performed on a 2 ml Source 15Q (GE Healthcare) column. Quercetinase was eluted with 0.2 M NaCl, concentrated by ultrafiltration, and stored in 50 mM Tris/HC (pH 8) at −80°C.

### Protein analysis

Molecular graphics was performed with the UCSF Chimera package, developed by the Resource for Biocomputing, Visualization, and Informatics at the University of California, San Francisco (supported by NIGMS P41-GM103311) [48]. Protein concentrations in cell-free extracts and CFPS reactions were estimated using the Bradford method as modified by Zor and Selinger [49]. Concentrations of electrophoretically pure QueD-Strep proteins were estimated from their absorbance at 280 nm, using the theoretical molar extinction coefficient of ε_280nm_ = 21,555 M^−1^ cm^−1^, calculated according to Pace *et al.* [50]. Metal contents of QueD preparations and buffer blanks were determined by inductively coupled plasma optical emission spectroscopy (ICP-OES), or by ICP-MS, performed by the Chemical Analysis Laboratory, Center for Applied Isotope Studies, University of Georgia (Athens, GA, USA). To prepare samples for metal analysis, the proteins were washed 3 times with 10-fold volumes of 50 mM Tris/HCl buffer (pH 8.0). Before use, the buffer was treated with Serdolit® Chelite® CHE (SERVA Electrophoresis, Heidelberg, Germany) to remove divalent cations. QueD activity was determined spectrophotometrically at 30°C by measuring quercetin consumption as described previously [3]. One unit (U) of QueD is defined as the amount of enzyme that catalyzes the conversion of 1 μmol of quercetin per minute at 30°C. Assays for the determination of apparent steady-state parameters of QueD for quercetin were performed in air-saturated buffer (50 mM Tris/HCl, pH 8.0) [3].

### Identification of organic reaction products

Samples for identifying the organic products of quercetin conversion by Co-QueD-E76H and Co-QueD were prepared and extracted as described previously [3]. LC/MS analysis of the extracts was performed on a Dionex™ Ultimate™ 3000 UHPLC system (Thermo Scientific), coupled to an amaZon speed ion trap mass spectrometer (Bruker). Products were separated with an Eurospher II 100-5 C18 column (Knauer GmbH, Berlin, Germany), using a linear gradient of 20% to 100% (over 45 min) methanol in 10 mM aqueous ammonium acetate, adjusted to pH 4.0 with acidic acid, at a flow rate of 0.4 ml min^−1^.

To analyze whether formate is formed from quercetin in a possible side reaction, 0.1 U ml^−1^ of Co-QueD or Co-QueD-E76H were incubated with 200 μM quercetin in 20 mM potassium phosphate buffer (pH 7.0) for 90 min. Subsequently, *Candida boidinii* formate dehydrogenase (Fluka, ~50 U ml^−1^) and NAD^+^ (40 mM) were added to final concentrations of 0.5 U ml^−1^ and 2 mM, respectively. Formation of NADH was monitored at 340 nm over 180 min. NAD^+^ solutions were always prepared freshly in 20 mM phosphate buffer (pH 7.0). For background correction, reference samples without formate dehydrogenase containing the QueD-quercetin reaction mixtures and NAD^+^ were monitored as well. Under these assay conditions, the detection limit of formic acid is in the μM range: 10 μM and 1 μM formic acid were detected with percentage deviations of ±12% and ±77%, respectively.

### Fluorescence titration

Dissociation constants *K*_D_ of enzyme-quercetin complexes were determined by fluorescence titrations, measuring intrinsic protein fluorescence in a Jasco FP-6500 spectrofluorometer. In an anaerobic glove box, degassed anoxic solutions of QueD protein were diluted in an anaerobic cuvette with N_2_-saturated buffer (50 mM Tris/HCl, pH 8.0, treated with Serdolit® Chelite® CHE) to a final concentration of approximately 1 μM. Small amounts of anoxic quercetin solution were added stepwise, mixed gently, and allowed to equilibrate at 30°C for 15 − 20 minutes. Upon excitation at 280 nm, the fluorescence intensities at λ_max_ = 340 nm were recorded after each titration step and plotted against the quercetin concentration. *K*_D_ values were determined from analysis of binding curves using the equation for a quadratic binding curve [51].

### Circular Dichroism (CD) spectroscopy

Far-UV CD spectra of proteins were recorded from 190 to 250 nm at 25°C with a J-815 circular dichroism spectrometer (Jasco), using a 0.1-cm path length cell, with a digital time integration of 1 s and a scan speed of 50 − 100 nm/min. Spectra were accumulated 7 − 10-fold. Protein samples were in 10 mM potassium phosphate buffer (pH 8.0). The protein concentration in the cuvette was adjusted to a final concentration of ~10 μM. All protein spectra were corrected by subtracting the CD spectrum of the buffer. To correct for errors such as slight differences in protein concentrations, spectra were scaled by normalizing each spectrum by ‖Θ‖ = (∫Θ(λ)^2^dλ)^1/2^ [52].

## Additional files

Additional file 1: Figure S1. Electrophoretic analysis of QueD proteins. Additional file 2: Table S1. Metal-related catalytic activity of QueD. Additional file 3: Table S2. Purification of QueD from *Streptomyces* sp. FLA. Additional file 4: Figure S2 and Table S3. Production of QueD-EGFP fusion protein by cell-free protein synthesis (CFPS), and quercetinase activity of QueD-EGFP in the CFPS reactions. Additional file 5: Figure S4. Verification of QueD synthesis by the CFPS system.

## Abbreviations

CD: Circular dichroism
CFPS: Cell-free protein synthesis
DTT: Dithiothreitol
FPLC: Fast protein liquid chromatography
HSAB: Hard-soft acids and bases
ICP-MS: Inductively coupled plasma mass spectroscopy
ICP-OES: Inductively coupled plasma optical emission spectroscopy
IPTG: Isopropyl-β-D-thiogalactopyranoside
LB: Lysogeny broth
LC/MS: Liquid chromatography coupled with mass spectroscopy
NADH: Nicotinamide adenine dinucleotide
QueD: Quercetinase of *Streptomyces* sp. FLA
U: Enzyme units
UV: Ultraviolet
