# Supplementary material for: Nickel quercetinase, a “promiscuous” metalloenzyme: metal incorporation and metal ligand substitution studies
Source: BMC Biochem. 2015 Apr 23;16:10. doi: 10.1186/s12858-015-0039-4 (PMC4416304; doi:10.1186/s12858-015-0039-4)
Supplement: Additional file 1: Figure S1. — Electrophoretic analysis of QueD proteins. [file 12858_2015_39_MOESM1_ESM.pdf]

Additional file 1:
Electrophoretic analysis of QueD proteins.

Figure S1. Strep-tagged Ni-QueD from recombinant *E. coli* (A), and QueD isolated from *Streptomyces* sp. FLA (B).

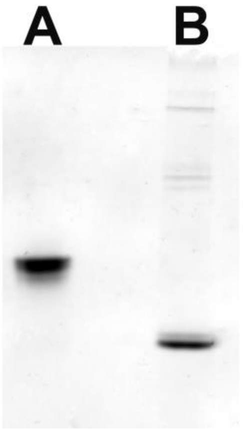

Native polyacrylamide gel electrophoresis (PAGE) was performed on a 10% polyacrylamide gel; the gel was stained with Coomassie blue. The identity of the protein isolated from the wild-type *Streptomyces* strain was verified by in-gel tryptic digestion and MALDI-MS analysis.
