# Supplementary material for: Nickel quercetinase, a “promiscuous” metalloenzyme: metal incorporation and metal ligand substitution studies
Source: BMC Biochem. 2015 Apr 23;16:10. doi: 10.1186/s12858-015-0039-4 (PMC4416304; doi:10.1186/s12858-015-0039-4)
Supplement: Additional file 2: Table S1. — Metal-related catalytic activity of QueD. [file 12858_2015_39_MOESM2_ESM.pdf]

Additional file 2:
Metal-related catalytic activity of QueD.

Table S1. Specific activities of *Streptomyces* quercetinase if completely loaded with a single metal species.
To extrapolate the specific activities of QueD proteins fully occupied with the indicated metal, it was assumed that the specific activity measured for proteins with mixed metal occupancy is a linear correlation of metal content and metal-related specific activity. 26 preparations of recombinant QueD with different metal occupancies were analyzed. The coefficient of determination R^2 is 0.848. Due to highly variable Mn contents and very low Zn contents in the preparations, errors are very large.

QueD form	Extrapolated metal-related specific activity ($U\ mg^{-1}$)	Relative error (%)
**Co-QueD**	28.5	49.8
**Cu-QueD**	$<< 10^{-10}$	∞
**Fe-QueD**	$<< 10^{-10}$	∞
**Mn-QueD**	46.3	84.5
**Ni-QueD**	183.3	8.4
**Zn-QueD**	79.4	75.7
