# Supplementary material for: Nickel quercetinase, a “promiscuous” metalloenzyme: metal incorporation and metal ligand substitution studies
Source: BMC Biochem. 2015 Apr 23;16:10. doi: 10.1186/s12858-015-0039-4 (PMC4416304; doi:10.1186/s12858-015-0039-4)
Supplement: Additional file 3: Table S2. — Purification of QueD from Streptomyces sp. FLA. [file 12858_2015_39_MOESM3_ESM.pdf]

Additional file 3:
Purification of QueD from *Streptomyces* sp. FLA.

Table S2: Enrichment of quercetinase QueD from *Streptomyces* sp. FLA.
Starting material was 35 g of wet biomass.

**Fraction**	**Activity (U)**	**Spec. activity (U/mg)**	**Enrichment (-fold)**	**Yield (%)**
Crude extract supernatant	216.2	0.11	1.00	100
Supernatant after ammonium sulfate precipitation	244.8	0.14	1.2	113
Phenyl-Sepharose CL-4B	99.5	0.71	6.3	46.1
Q Sepharose Fast Flow	31.1	6.15	54.0	14.4
Source 15Q	8.0	48.63	427.7	3.7
