# Supplementary material for: Nickel quercetinase, a “promiscuous” metalloenzyme: metal incorporation and metal ligand substitution studies
Source: BMC Biochem. 2015 Apr 23;16:10. doi: 10.1186/s12858-015-0039-4 (PMC4416304; doi:10.1186/s12858-015-0039-4)
Supplement: Additional file 4: Figure S2 and Table S3. — Production of QueD-EGFP fusion protein by cell-free protein synthesis (CFPS), and quercetinase activity of QueD-EGFP in the CFPS reactions. [file 12858_2015_39_MOESM4_ESM.pdf]

Additional file 4:

Production of QueD-EGFP fusion protein by cell-free protein synthesis (CFPS), and quercetinase activity of QueD-EGFP in the CFPS reactions.

Figure S2: Cell-free synthesis of QueD-EGFP in the presence of metal ions.

Average fluorescence emission of the fusion protein QueD-EGFP in cell-free protein synthesis (CFPS) reactions which contained the indicated M^{2+} ions at varying concentrations (A−E), and without additional metal ions (F). Fluorescence emission at 510−570 nm upon excitation at 490 nm was determined every 20 min in a Glomax®-multi+ microplate reader (Promega). RFU, relative fluorescence units.

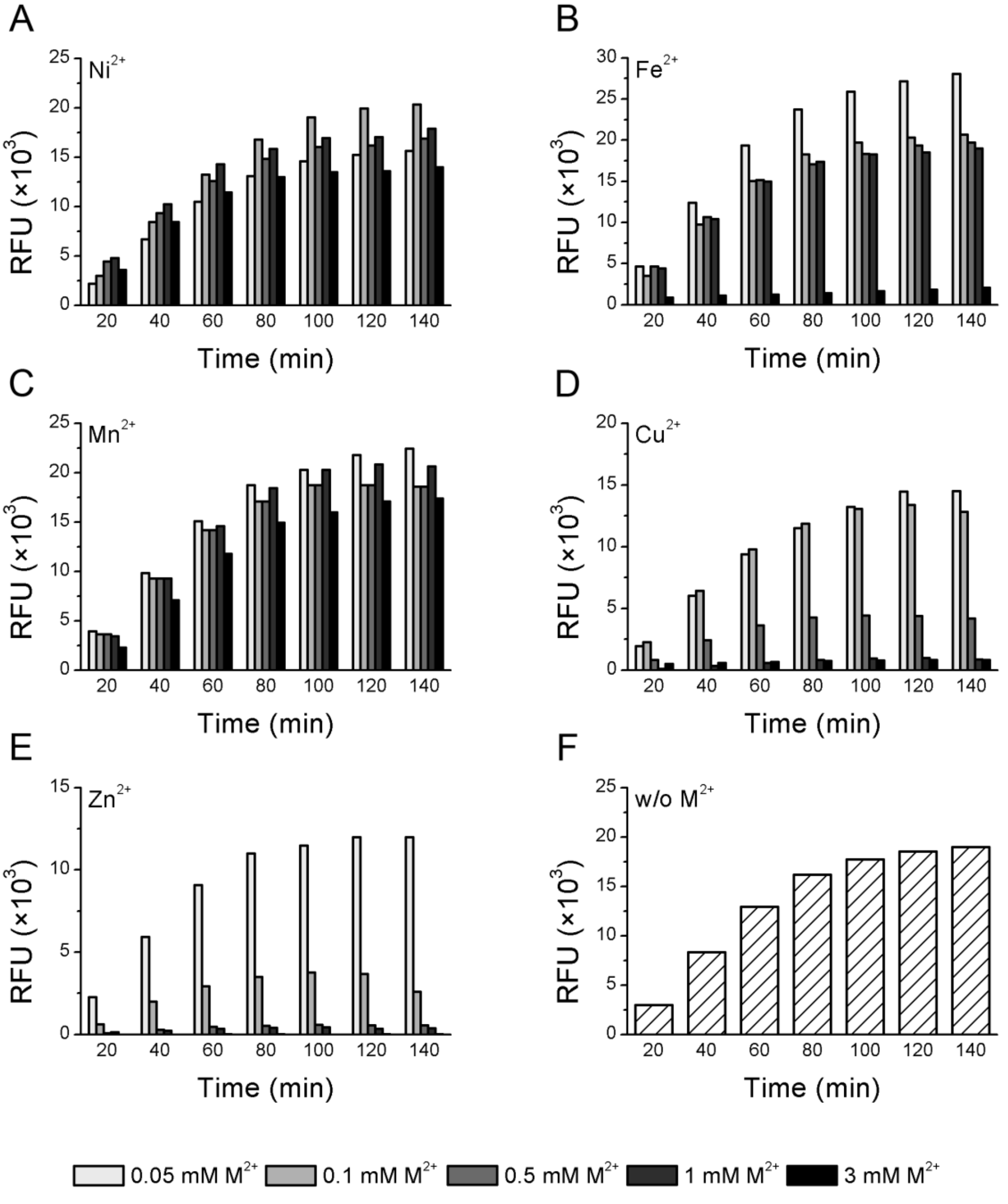

Table S3: Specific quercetinase activity of QueD-EGFP fusion proteins in CFPS reactions.

Concentration of M^{2+} in CFPS (mM)	Specific activity (mU mg^{-1}) after CFPS in the presence of: [a]				
	Ni^{2+}	Mn^{2+}	Fe^{2+}	Cu^{2+}	Zn^{2+}
0.05	75	3.2	1.7	0.7	0.6
0.1	194	4.2	1.4	0.7	0.6
0.5	906	16.7	1.8	b.d.[b]	0.9
1.0	1580	25.9	4.3	2.1	b.d.
3.0	1123	32.8	1.5	1.4	b.d.

[a] Quercetinase activity was measured after 3 h of incubation directly in the CFPS system, which contains *E. coli* cell extracts (protein concentrations in the CFPS assays were in the range of 20−40 mg ml^{-1}). The specific quercetinase activity of QueD-EGFP after CFPS performed without M^{2+} supplementation was 1.8 mU mg^{-1}. All activity data were corrected for spontaneous quercetin oxidation in presence of the same concentration of the respective metal ion.
