# Supplementary material for: Nickel quercetinase, a “promiscuous” metalloenzyme: metal incorporation and metal ligand substitution studies
Source: BMC Biochem. 2015 Apr 23;16:10. doi: 10.1186/s12858-015-0039-4 (PMC4416304; doi:10.1186/s12858-015-0039-4)
Supplement: Additional file 5: Figure S4. — Verification of QueD synthesis by the CFPS system. [file 12858_2015_39_MOESM5_ESM.pdf]

Additional file 5: Verification of QueD synthesis by the CFPS system.

Figure S4. Western blot of denaturing polyacrylamide gel of QueD proteins produced by cell-free protein synthesis.

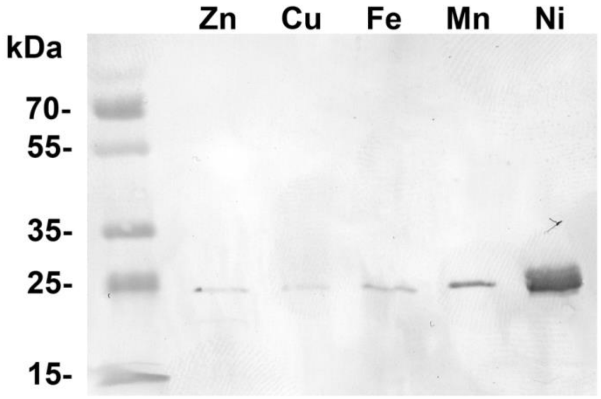

Figure S4. QueD proteins were produced by cell-free protein synthesis in the presence of various M^{2+} ions (1 mL scale) and purified by Strep-tactin affinity chromatography. The metal ion added to the cell-free protein synthesis reaction is indicated above the lanes. Denaturing (sodium dodecyl sulfate) PAGE was performed as described by Laemmli [1], using an overall acrylamide concentration of 12.5% and a crosslinker concentration of 2.6% in the separating gel. Semi-dry electroblotting was performed according to Towbin *et al*. [2]. Immunodetection of Strep-tagged proteins was carried out with primary Anti-Strep IgG1 mouse antibodies (IBA) and secondary Anti-mouse IgG antibodies fused to alkaline phosphatase (Sigma-Aldrich).

1. Laemmli UK: **Cleavage of structural proteins during the assembly of the head of bacteriophage T4**. *Nature* 1970, **227:**680–685.
2. Towbin H, Staehelin T, Gordon J: **Electrophoretic transfer of proteins from polyacrylamide gels to nitrocellulose sheets: procedure and some applications**. *Proc Natl Acad Sci USA* 1979, **76:**4350–4354.
